# Ser9 phosphorylation of GSK-3β promotes aging in the heart through suppression of autophagy

**DOI:** 10.20517/jca.2021.13

**Published:** 2021-08-23

**Authors:** Yanbin Chen, Yasuhiro Maejima, Akihiro Shirakabe, Takanobu Yamamoto, Yoshiyuki Ikeda, Junichi Sadoshima, Peiyong Zhai

**Affiliations:** 1 Department of Respiratory Medicine, The First Affiliated Hospital of Soochow University, Soochow 215000, Jiangsu, China.; 2 Department of Cardiovascular Medicine, Tokyo Medical and Dental University, Tokyo 113-8510, Japan.; 3 Division of Intensive Care Unit, Nippon Medical School Chiba Hokusoh Hospital, Chiba 270-1694, Japan.; 4 Department of Cardiovascular Medicine and Hypertension, Graduate School of Medicine, Kagoshima University, Kagoshima 890-8580, Japan.; 5 Department of Cell Biology and Molecular Medicine, Rutgers New Jersey Medical School, Newark, NJ 07103, USA.

**Keywords:** GSK-3, autophagy, Ulk1, aging, senescence

## Abstract

**Introduction:**

Glycogen synthase kinase-3β (GSK-3β) is a serine/threonine kinase and a negative regulator of cardiac hypertrophy. Phosphorylation of GSK-3β at Ser9 negatively regulates its kinase activity. The role of GSK-3β in cardiac aging remains poorly understood.

**Aim:**

The study aimed to elucidate the role of GSK-3β Ser9 phosphorylation in mediating cardiac aging and the underlying mechanism.

**Methods and Results:**

Phosphorylation of GSK-3β at Ser9 and the levels of β-catenin and Mcl-1 were increased in the mouse heart during aging, suggesting that GSK-3β is inactivated during aging in the heart. Age-induced cardiac hypertrophy, fibrosis, left ventricular dysfunction, and increases in cardiomyocyte apoptosis and senescence were all attenuated in constitutively active GSK-3β^S9A^ knock-in (KI) mice compared to littermate wild type mice. Although autophagy is inhibited in the heart during aging, KI of GSK-3β^S9A^ reversed the age-associated decline in autophagy in the mouse heart. GSK-3β directly phosphorylates Ulk1, a regulator of autophagy, at Ser913, thereby stimulating autophagy in cardiomyocytes. Ulk1Ser913A KI mice exhibited decreased autophagic flux and increased senescence in cardiomyocytes.

**Conclusion:**

Our results suggest that GSK-3β is inactivated during aging through Ser9 phosphorylation, which in turn plays an important role in mediating cardiac aging. GSK-3β promotes autophagy through phosphorylation of Ulk1 at Ser913, which in turn prevents aging in the heart.

## One sentence summary

Ser9 phosphorylation of GSK-3β promotes aging in the heart at least in part through decreases in Ulk1 phosphorylation at Ser913 and suppression of autophagy.

## INTRODUCTION

Aging is characterized by a progressive accumulation of damaged macromolecules and organelles in postmitotic cells, leading to cellular dysfunction and increased vulnerability to stress^[1,2]^. The aging-associated structural changes in the heart result in an increase in cardiomyocyte size, a decrease in the cardiomyocyte number, and an increase in matrix connective tissue. The functional changes lead to a decline in diastolic function, manifested by a reduced left ventricular (LV) early diastolic filling rate, whereas systolic function is relatively maintained at rest, although there is a decrease in maximum ejection fraction^[3]^. Aging mice exhibit cardiac hypertrophy, fibrosis, apoptosis, and both systolic and diastolic dysfunction in the heart^[4,5]^.

The prevalence of heart failure increases dramatically in aging populations^[6]^. The process of macroautophagy (referred to as autophagy hereafter) has been recognized to play a direct role in protecting the heart against heart failure^[7]^. Autophagy is a housekeeping mechanism by which the cell degrades dysfunctional or unnecessary molecules and organelles^[8]^. Both the formation and the elimination of autophagosomes decrease with aging^[9]^, and the impairment of autophagic flux results in premature senescence^[10]^. The suppression of autophagy in mice is associated with aging-related cardiac abnormalities^[11,12]^. On the other hand, the enhancement of autophagic flux is associated with anti-aging phenotypes in mice^[13]^. We and others have shown that autophagy is suppressed in aging hearts^[5,11,14]^. However, the molecular mechanism through which aging induces a decline in autophagy remains unclear. In mammals, autophagy is thought to be initiated by a kinase complex containing the serine/threonine kinases Uncoordinated-51-like kinase 1 (Ulk1) and Ulk2, mammalian autophagy-related (Atg) 13, and focal adhesion kinase family interacting protein of 200 kD (FIP200)^[15,16]^. The initiation step of autophagosome formation is essential for autophagy to occur and is highly regulated. Among the components of the initiation complex, Ulk1 is a key regulator of autophagy initiation^[15–18]^. Currently, whether the function of Ulk1 is altered during aging is unknown.

GSK-3β is a ubiquitously expressed serine/threonine kinase that regulates a wide variety of cellular functions, including gene expression, metabolism, cell growth, hypertrophy, and apoptosis, in cardiomyocytes. GSK-3β is active under resting conditions, and its activity is inhibited by phosphorylation at Ser9 by upstream kinases (e.g., Akt). Ser9 phosphorylation of GSK-3β is augmented in the liver tissue of 12- and 18-month-old rats^[9]^ and in stress-induced cellular senescence^[19]^, and it is highly elevated in the hearts of old rats (20–24 months old)^[20,21]^. Furthermore, GSK-3 inhibition by a small molecule inhibitor, SB415286, induces cellular senescence^[19]^. The decline in the expression of GSK-3β with age causes aging-related loss of the regenerative capacities of the mouse liver^[22]^. Together, these studies suggest that GSK-3β inhibition may play an important role in aging. However, there is a lack of direct evidence delineating whether Ser9 phosphorylation of GSK-3β is directly involved in aging in the heart. In addition, the mechanisms through which GSK-3β modulates aging remain to be elucidated.

In this study, we hypothesized that GSK-3β regulates myocardial aging through modulation of Ulk1, a key regulator of autophagy. We investigated the role of GSK-3β phosphorylation at Ser9 during cardiac aging, using GSK-3β^S9A^ knock-in (βKI) mice, in which GSK-3β is constitutively active. We also investigated the role of GSK-3β in regulating autophagy during aging and the underlying molecular mechanisms.

## METHODS

### Genetically modified mice

βKI mice (C57BL/6 background) were provided by Dr. D. R. Alessi (University of Dundee, Dundee, UK)^[23]^. The basal characterization of βKI mice has been described previously^[23]^. The cardiac phenotype of the homozygous βKI mice is not significantly different from that of wild type (WT) mice at 3 months of age. Systemic Ulk1S913A KI mice were generated using the Crispr Cas9 technique at the Rutgers Genome Editing Shared Resources facility. All animal experiments were approved by the Institutional Animal Care and Use Committee of the New Jersey Medical School, Rutgers, The State University of New Jersey. The animal use in this study also conformed to the Guide for the Care and Use of Laboratory Animals published by the United States National Institutes of Health (8th edition, 2011). All animal experiments use both male and female mice. The investigators were blinded to the genotype groups during the experiments, but randomization was not needed for the animal studies because there were no further treatment allocations.

### *In vitro* kinase assay and mass spectrometry

To examine phosphorylation of Ulk1 by GSK-3β, an *In vitro* kinase assay was carried out as described^[24]^. Briefly, recombinant active GSK-3β (10 ng, 25 μL) (Millipore) was incubated with GST-tagged human Ulk1^K46R^ (1 μg) in a kinase buffer (50 mM HEPES (pH 7.4), 15 mM MgCl_2_ and 200 μM sodium vanadate containing 100 μM ATP or 5 μM ATP and 10 μCi [γ−^32^P] ATP per reaction) at 30 °C for 30 min. Phosphorylated proteins were separated by SDS-PAGE and analyzed by autoradiography. Mass spectrometry was performed by the Proteomic Core Facility at the New Jersey Medical School after a cold *In vitro* kinase assay.

### Primary culture of neonatal rat ventricular cardiomyocytes

Primary cultures of ventricular cardiomyocytes were prepared from one-day-old Charles River Laboratories (Crl)/Wistar Institute (WI) BR-Wistar rats (Harlan Laboratories, Somerville, NJ, USA) and maintained in culture as described previously^[25]^. A cardiomyocyte-rich fraction was obtained by centrifugation through a discontinuous Percoll gradient.

### Echocardiography

Two-dimension (short-axis)-guided M-mode measurements (at the level of the papillary muscles) of LV internal diameter were taken from three or more beats and averaged. LV end-diastolic dimension (LVEDD) was measured at the time of the apparent maximal LV diastolic dimension, whereas LV end-systolic dimension (LVESD) was measured at the time of the most anterior systolic excursion of the posterior wall. Systolic function was quantified as LV ejection fraction (LVEF), estimated from LV dimensions by the cubed method as follows: LVEF = (LVEDD^3^ - LVESD^3^)/LVEDD^3^.

### Pressure-volume loop analysis

To measure cardiac systolic and diastolic function, pressure-volume (P-V) loop analysis was carried out as previously described^[26,27]^. The end-systolic (ES) elastance and chamber stiffness constant were calculated from P-V loops obtained by altering preload through temporary occlusion of the inferior vena cava.

### Histological analysis and evaluation of apoptosis in tissue sections

Histological analyses of LV sections were conducted as described previously^[28]^. Interstitial fibrosis was evaluated by PASR staining. The positively stained (red) fibrotic area was expressed as a percentage of total area. Cardiomyocyte cross-sectional area was determined from wheat germ agglutinin-rhodamine (Vector Laboratories)-stained cardiac tissue sections. The mean cardiomyocyte cross-sectional area was calculated for each animal, and the group mean was then calculated for each group. DNA fragmentation was detected in situ using TUNEL. Nuclear density was determined by manual counting of DAPI (4’,6-diamidino-2-phenylindole)-stained nuclei in 20 fields from each animal using the 40× objective and of TUNEL-positive nuclei in the same fields using the same power objective.

### Immunoblotting analysis

Cardiac tissue homogenates were prepared from the LV apex. Cell lysates were prepared from primary cultures of rat ventricular myocytes 24–72 h after adenovirus infection and 4 h after glucose deprivation. Both the homogenates and the cell lysates were prepared in a radioimmunoprecipitation assay (RIPA) buffer containing 150 mM NaCl, 1% Triton-X 100, 0.5% sodium deoxycholate, 0.1% sodium dodecyl sulfate, and 50 mM Tris, pH 8.0 and supplemented with a protease inhibitor cocktail (Sigma), 5 mM NaF, and 1 mM sodium orthovanadate. We used antibodies against GSK-3α [Cell Signaling Technology (CST), #9338], GSK-3β (CST, #9315), phospho-GSK-3α (S21) (CST, #9316), phospho-GSK-3β (S9) (CST, #9336), β-catenin (CST, #9582), MCL-1 (Rockland, #600–401-394), p62 (Abcam, #ab91526), LC3 (MBL, #M186–3), phospho-H2AX (S139) (CST, #9718), H2AX (CST, #7631), GAPDH (CST, #2118), and α-tubulin (Sigma, #T6199).

### Generation of Ulk1 Ser913 phosphospecific antibody

The phosphospecific antibody against Ulk1 Ser913 was generated by GenScript USA Inc (Piscataway, New Jersey) using the peptide LS{pSER}GLQTAIDQIRAC as an immunogen. The phosphospecific antibody was purified from antiserum collected after the third immunization in rabbit.

### Statistical analysis

The data are presented as mean ± SEM and were first analyzed using analysis of variance (ANOVA). If significant differences were observed, post hoc analysis was performed using the *t*-test with Bonferroni correction. A *P* value less than 0.05 was considered significant.

## RESULTS

### Phosphorylation of GSK-3β was increased in aged mouse hearts

**Figure 1.**
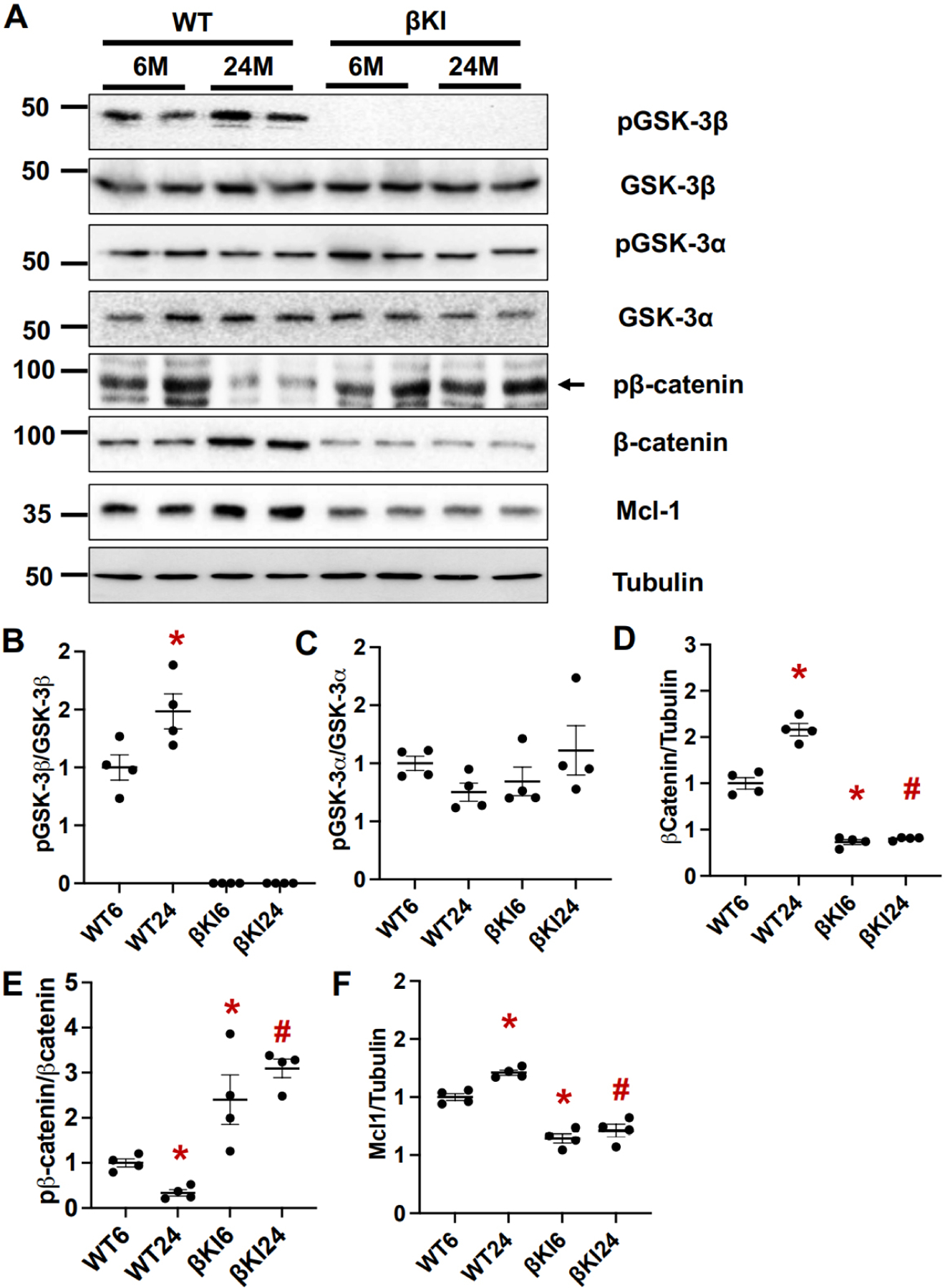
GSK-3β and GSK-3α in the aged heart. (A) Immunoblots of phospho(S9)-GSK-3β, GSK-3β, phospho(S21)-GSK-3α, GSK-3α, β-catenin, myeloid cell leukemia 1 (Mcl-1), and tubulin in left ventricular homogenates from WT and homozygous GSK-3β^S9A^ knock-in mice (βKI) at 6 and 24 months (M) of age. The results of two experiments (out of four) are shown. (B) Relative band density of pGSK-3β/GSK-3β. *P < 0.05 *vs.* WT 6M (WT6). WT24, WT 24M; βKI6, βKI 6M; βKI24, βKI 24M. (C) Relative band density of pGSK-3 α/GSK-3α. (D) Relative band density of β-catenin/tubulin. *P < 0.05 *vs.* WT6, ^#^*P* < 0.05 *vs.* WT24. (E) Relative band density of phospho-β-catenine/β-catenin. *P < 0.05 *vs.* WT6, ^#^*P* < 0.05 *vs.* WT24. (F) Relative band density of Mcl-1/tubulin. *P < 0.05 *vs.* WT6, ^#^ *P* < 0.05 *vs.* WT24. In (B-F), *n* = 4 in each group. WT: Wild type.

To investigate the role of GSK-3β in cardiac aging, we first evaluated the levels of total and Ser9 phosphorylated GSK-3β in mouse hearts at 6 and 24 months of age. Ser9 phosphorylated GSK-3β, an inactive form of GSK-3β, normalized by the total level of GSK-3β was increased in the heart at 24 months compared to 6 months of age [Figure 1A and B]. The levels of GSK-3β substrates, including β-catenin and myeloid cell leukemia 1 (Mcl-1), known to be increased when GSK-3β is inactivated^[26]^, were also greater in the heart at 24 months than at 6 months of age [Figure 1A, D, E]. In contrast, phosphorylation of GSK-3α at Ser21 normalized by total GSK-3α was not significantly different in the heart between 6 and 24 months of age, consistent with a previous report^[12]^ [Figure 1A and C]. These results suggest that GSK-3β is phosphorylated at Ser9 and inactivated, whereas Ser21 phosphorylation of GSK-3α is not altered in aged hearts. As expected, in βKI hearts, GSK-3β phosphorylation was completely abolished and neither β-catenin nor Mcl-1 was upregulated at 24 months compared to 6 months of age. These results suggest that βKI mice are useful for the investigation of the role of endogenous GSK-3β phosphorylation at Ser9 during cardiac aging.

### Phosphorylation of GSK-3β at Ser9 promotes LV dysfunction and pathological changes in the heart during aging

**Figure 2.**
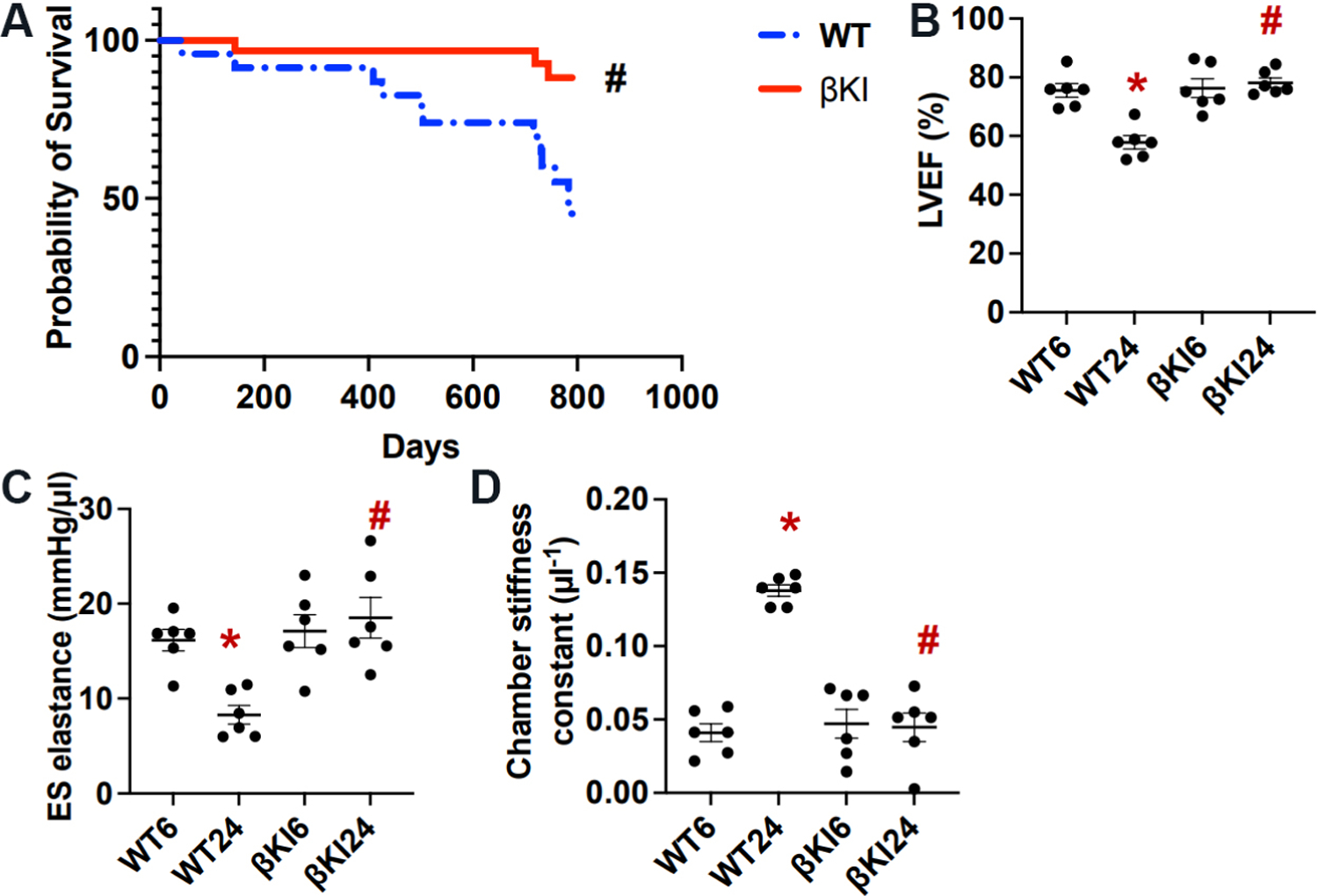
GSK-3β^S9A^ knock-in (βKI) preserves cardiac function and improves survival during aging. (A) Survival curves for WT mice (*n* = 21) and homozygous βKI mice (*n* = 20). Statistical analysis was conducted using the Gehan-Breslow-Wilcoxon test. ^#^*P* < 0.05 βKI *vs.* WT. (B) LV ejection fraction (EF) in WT and homozygous βKI mice. *P < 0.05 *vs.* WT six months (WT6); ^#^*P* < 0.05 *vs.* WT 24 months (WT24). (C) LV end-systolic (ES) elastance in WT and homozygous βKI. *P < 0.05 *vs.* WT6; ^#^*P* < 0.05 *vs.* WT24. (D) LV chamber stiffness constant in WT and homozygous βKI. *P < 0.05 *vs.* WT6; ^#^*P* < 0.05 *vs.* WT24. LV: Left ventricular; WT: wild type.

We next investigated how the aging-induced increase in phosphorylation of GSK-3β at Ser9 affects the survival of control and βKI mice up to 790 days. A Kaplan-Meier analysis showed that βKI mice exhibit a significantly higher survival than WT mice [Figure 2A].

We then investigated the role of the aging-induced increases in phosphorylation of GSK-3β at Ser9 in regulating cardiac function. We found that aging facilitates declines in LV systolic function, evaluated by measuring LVEF [Figure 2B] and ES elastance [Figure 2C], and LV diastolic function, evaluated by measuring chamber stiffness [Figure 2D]. Both LVEF and ES elastance in WT mice were significantly lower and the chamber stiffness constant was significantly higher in WT mice at 24 months than at 6 months of age, suggesting that both systolic and diastolic dysfunction develop in WT mice during aging. In contrast, none of these three parameters differed significantly at either 6 or 24 months of age in βKI mice from those in WT mice at six months of age [Figure 2B and D]. These results suggest that age-dependent increases in Ser9 phosphorylation of endogenous GSK-3β play an important role in mediating systolic and diastolic cardiac dysfunction in mice.

**Figure 3.**
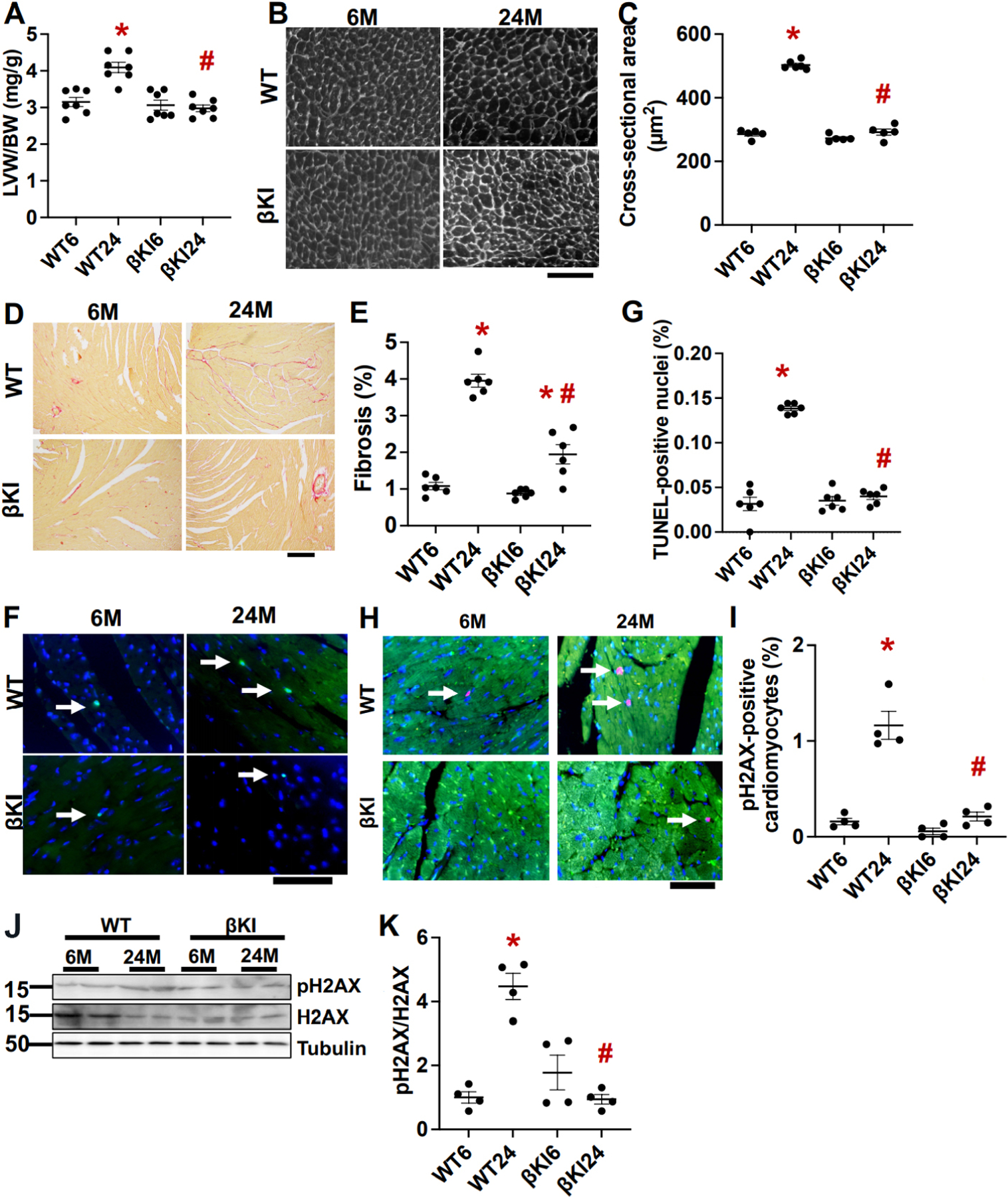
GSK-3β^S9A^ knock-in (βKI) attenuates cardiac hypertrophy, fibrosis, apoptosis, and senescence during aging. (A) Left ventricular weight (LVW)/body weight (BW) in WT and homozygous βKI. *P < 0.05 *vs.* the same genotype at six months (6); ^#^*P* < 0.05 *vs.* WT24. (B) Wheat germ agglutinin staining of cardiac tissue sections from WT and homozygous βKI. Scale bar = 100 μm. (C) Cardiomyocyte cross-sectional area of WT and homozygous βKI. *P < 0.05 *vs.* WT6; ^#^*P* < 0.05 *vs.* WT24. (D) Picric acid Sirius red (PASR) staining in WT and homozygous βKI mice. Scale bar = 200 μm. (E) Percent cardiac fibrosis out of total image area in WT and homozygous βKI. *P < 0.05 *vs.* the same genotype at six months (6); ^#^*P* < 0.05 *vs.* WT24. (F) TUNEL and DAPI staining in WT and homozygous βKI. Arrows point to positive TUNEL-stained nuclei. Scale bar = 100 μm. (G) Percent of TUNEL-positive nuclei in WT and homozygous βKI mice. *P < 0.05 *vs.* WT6; ^#^*P* < 0.05 *vs.* WT24. (H) Senescence marker pH2AX in WT and βKI mice at 6 and 24 months old (M). Green, cardiac troponin T. Red, pH2AX; Blue, DAPI. Arrows point to positively stained nuclei. Scale bar = 20 μm. (I) The percent of pH2AX-positive cardiomyocytes in WT and βKI. *P < 0.05 *vs.* WT at six months (WT6); ^#^*P* < 0.05 *vs.* WT at 24 months (WT24). (J) Immunoblots of pH2AX, H2AX, and α-tubulin in left ventricular homogenates of WT and βKI. (K) Relative band density of pH2AX/H2AX. *P < 0.05 *vs.* WT6; ^#^*P* < 0.05 *vs.* WT24. WT: Wild type.

Cardiac hypertrophy, indicated by increases in left ventricular weight/body weight and left ventricular cardiomyocyte cross sectional area, was significantly greater at 24 months than at 6 months of age in WT mice, whereas aging-induced cardiac hypertrophy was abolished in βKI mice [Figure 3A–C]. Although both cardiac interstitial fibrosis, as evaluated with picrosirius red staining, and cardiomyocyte apoptosis, as evaluated with TUNEL staining, were greater at 24 months than at 6 months of age in WT mice, the aging-induced increase in fibrosis was significantly attenuated and that in apoptosis was fully abolished in βKI mice [Figure 3D–G]. These results suggest that increases in Ser9 phosphorylation of endogenous GSK-3β play an important role in mediating aging-induced pathological changes, including hypertrophy, fibrosis, and apoptosis, in the mouse heart.

Senescent cells promote aging in the heart through the senescent associated secretory phenotype in cardiomyocytes. Senescence in cardiomyocytes, as evaluated with pH2AX immunostaining, was greater at 24 months than at 6 months of age in WT mice, whereas aging-induced increases in senescent cardiomyocytes were abolished in βKI mice [Figure 3H–K]. These results suggest that phosphorylation of GSK-3β at Ser9 promotes senescence in the heart.

### Aging-induced decreases in autophagy were suppressed in βKI mice

**Figure 4.**
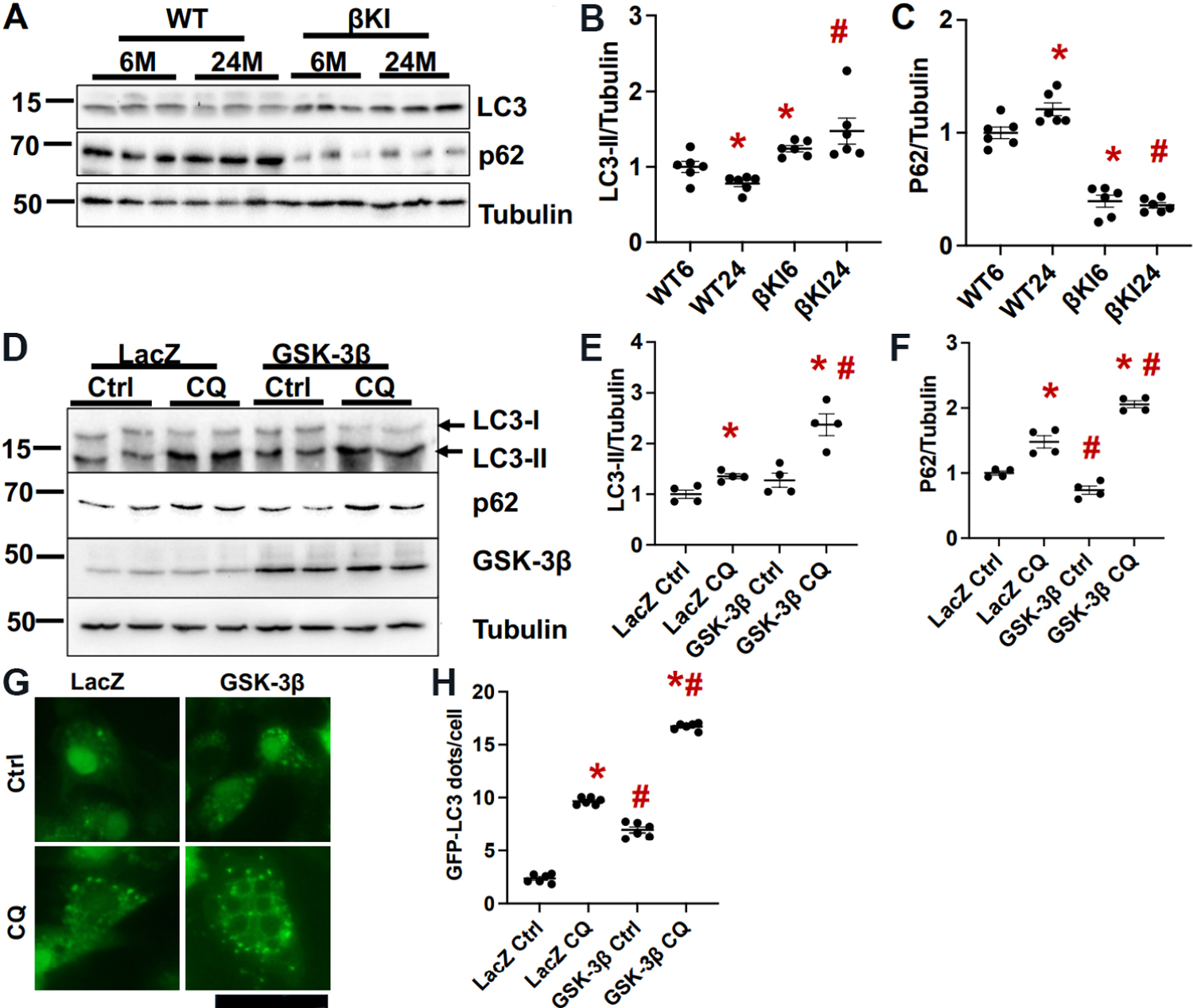
GSK-3β^S9A^ knock-in (βKI) attenuates aging-induced decreases in autophagy. (A) Immunoblots of LC3, p62, and α-tubulin in left ventricular homogenates of WT and homozygous βKI mice at 6 and 24 months (M). (B) Relative band density of LC3-II/tubulin. (C) Relative band density of p62/tubulin. In (B, C), WT6, WT 6M; βKI6, βKI 6M; WT24, WT 24M; βKI6, βKI 6M. The relative band density of WT6 was designated as 1. *P < 0.05 *vs.* WT6; ^#^*P* < 0.05 *vs.* WT of the same age. *n* = 6 in each group. (D) Immunoblots of LC3, p62, and tubulin in cardiomyocytes transduced with LacZ or GSK-3β, with or without chloroquine treatment (CQ, 20 µM) for 4 h. The results of two experiments (out of four) are shown. (E) Relative band density of LC3-II/tubulin. (F) Relative band density of p62. In (E, F), the relative band density in LacZ-transduced cells treated with vehicle was designated as 1. ^#^*P* < 0.05 *vs.* LacZ of the same treatment; *P < 0.05 *vs.* vehicle control (Ctrl) with the same virus infection. *n* = 4 in each group. (G) GFP-LC3 dots in cardiomyocytes transduced with GFP-LC3 and LacZ or GSK-3β, with or without chloroquine treatment (CQ, 20 µM) for 4 h. The number of GFP-LC3 dots is quantified in (H). In each experimental repeat, 20 cardiomyocytes were counted. ^#^*P* < 0.05 *vs.* LacZ of the same treatment; *P < 0.05 *vs.* vehicle control (Ctrl) with the same virus infection. WT: Wild type.

Autophagy is decreased by aging in various organs, which in turn contributes to aging-induced dysfunction in those organs^[14]^. We showed previously that autophagic flux in the heart is decreased by aging^[5]^. The level of LC3II was lower, whereas that of p62 was higher in the WT mouse heart at 24 months than at 6 months of age, consistent with our previous results. In βKI mouse hearts at 24 months of age, however, the level of LC3II was higher and that of p62 was lower than in the WT mouse heart [Figure 4A–C]. These results are consistent with the notion that aging induces inhibition of autophagy in the heart.

Overexpression of WT GSK-3β in cultured cardiomyocytes increased LC3II and decreased p62 at baseline compared to LacZ expression. However, chloroquine treatment enhanced accumulation of LC3II and p62 in the presence of GSK-3β overexpression compared to LacZ [Figure 4D–F]. GSK-3β also increased the number of GFP-LC3 dots in cardiomyocytes both at baseline and after chloroquine treatment [Figure 4G and H]. These results suggest that GSK-3β promotes autophagy and autophagic flux in cardiomyocytes in a cell autonomous manner.

### GSK-3β directly phosphorylates Ulk1 at Ser913

**Figure 5.**
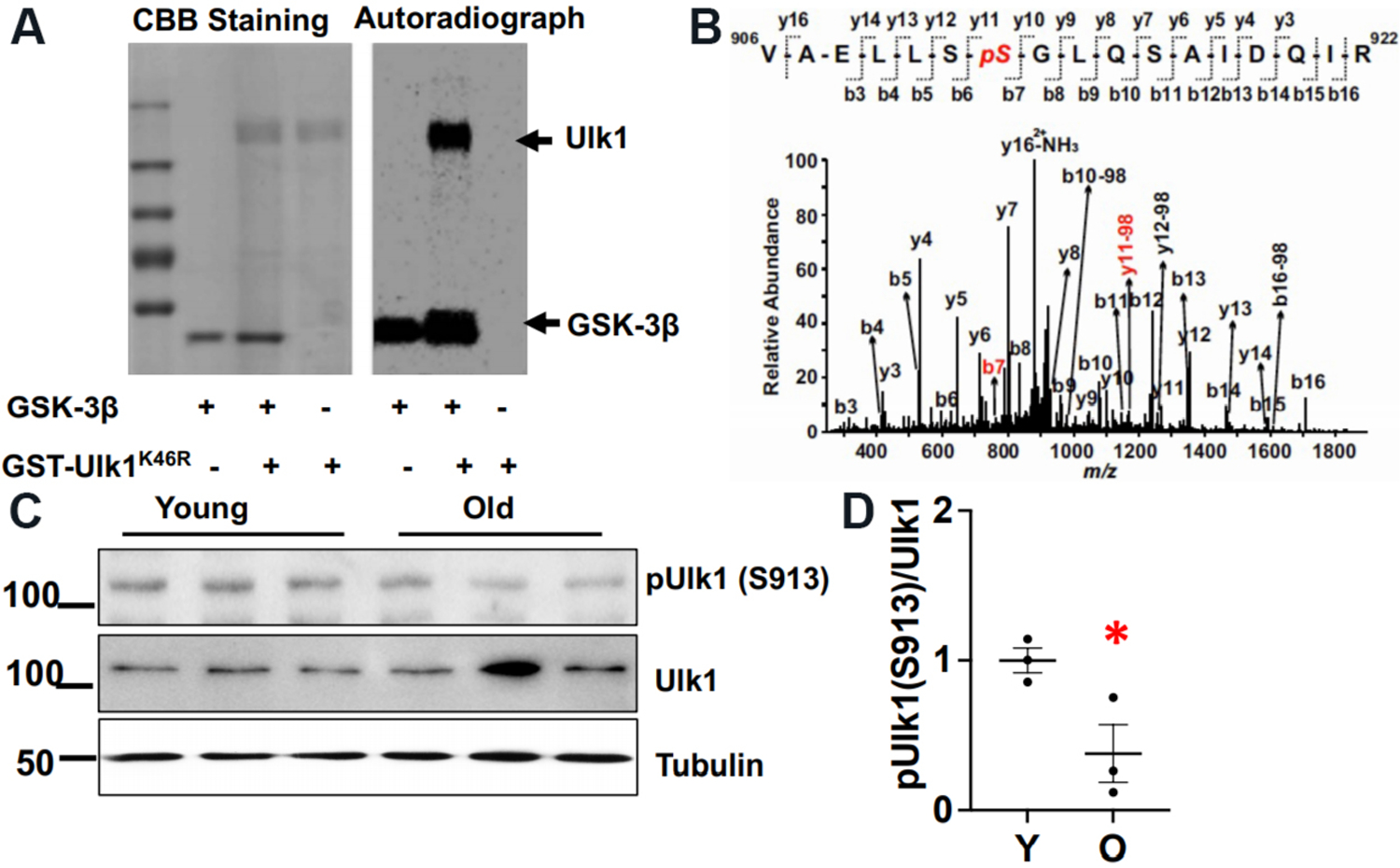
GSK-3β phosphorylates Ulk1. (A) Coomassie brilliant blue (CBB) staining and autoradiography after *in vitro* kinase assay using active recombinant GSK-3β as a kinase and GST- Ulk1^K46R^, a kinase inactive mutant of human Ulk1, as a substrate. (B) Mass spectrum showing the phosphorylation site (Ser912) in the human Ulk1 sequence. (C) Phosphorylation of Ulk1 at Ser913 in young (Y) and old (O) wild type mice. An immunoblot of GAPDH is shown as an internal control. The bar graph shows the relative band density of pUlk1/Ulk1. The pUlk1/Ulk1 ratio in young mice was designated as 1. *P < 0.05 *vs.* young.

To identify the downstream molecule through which GSK-3β regulates autophagy, we searched for a consensus amino acid sequence for GSK-3β-mediated phosphorylation in proteins involved in autophagy and discovered that Ulk1, a mammalian Atg1 kinase, has that consensus amino acid sequence. To test whether GSK-3β directly phosphorylates Ulk1, we performed *in vitro* kinase assays using recombinant GSK-3β as the kinase and human Ulk1^K46R^, a kinase inactive mutant, as the substrate. Autoradiography after the *in vitro* kinase assay showed that GSK-3β directly phosphorylates Ulk1 [Figure 5A]. Mass spectrometry analysis identified the phosphorylation site as Ser912 (corresponding to Ser913 in the mouse and rat sequences) [Figure 5B]. The extent of Ulk1 phosphorylation at Ser913/total Ulk1 was greater in mouse hearts at 6 months of age than at 24 months of age, as determined by immunoblot analyses with anti-Ser913 phosphorylated Ulk1-specific antibody [Figure 5C].

### Phosphorylation of Ulk1 at Ser913 may stimulate autophagy in the hearts

**Figure 6.**
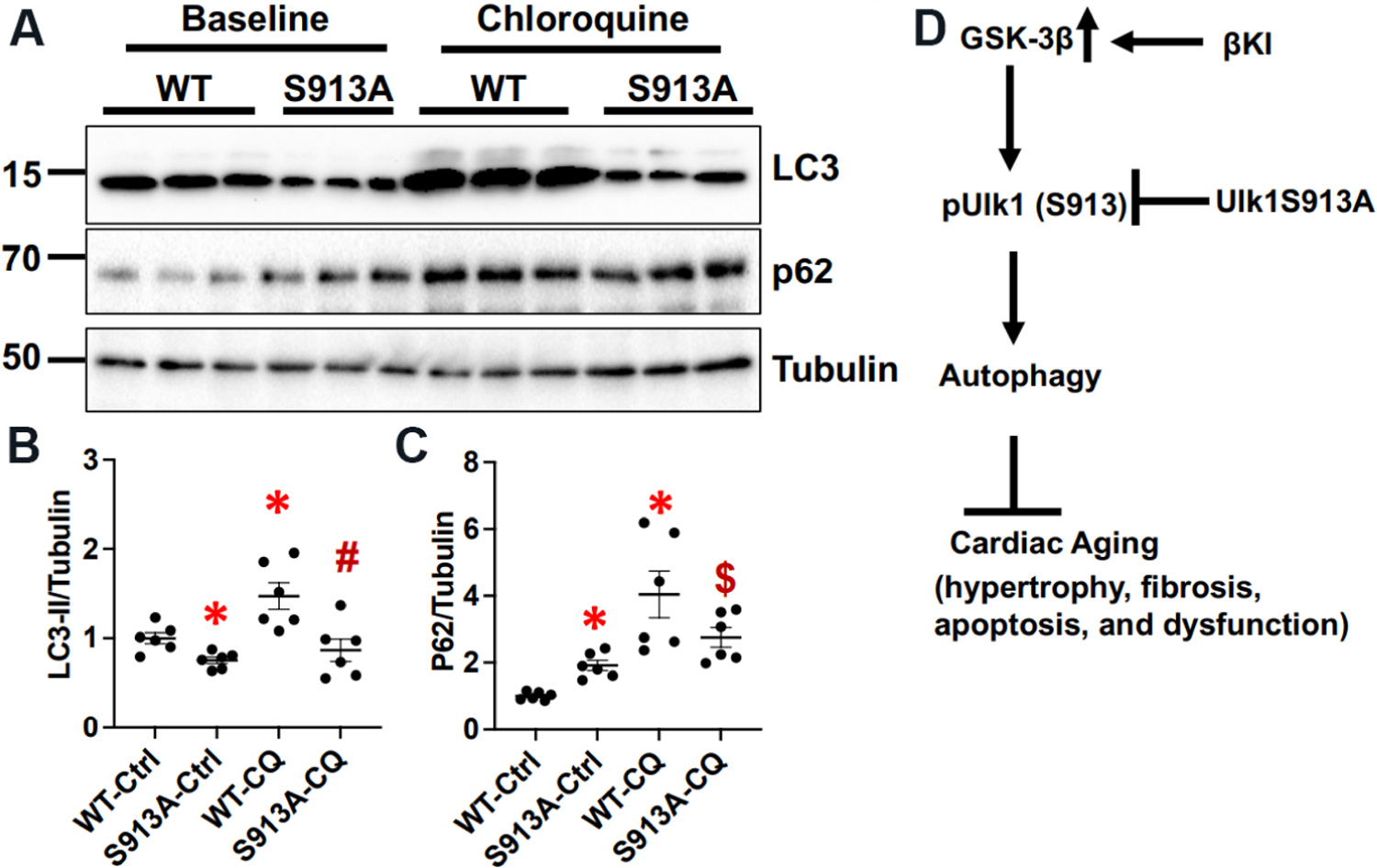
Ulk1S913A knock-in (KI) inhibits autophagy in the heart. Ulk1S913A KI and wild type (WT) mice were euthanized at six months of age. (A) Immunoblotting of LC3, p62, and α-tubulin in Ulk1S913A KI mouse hearts at baseline and 4 h after chloroquine (CQ) treatment. (B) The relative band density of LC3-II. The LC3-II/Tubulin in the WT control (Ctrl) group were designated as 1. *P < 0.05 *vs.* WT-Ctrl; ^#^*P* < 0.05 *vs.* WT-CQ. (C) The relative band density of p62. The p62/Tubulin in the WT-Ctrl group were designated as 1. *P < 0.05 *vs.* WT-Ctrl; ^$^*P* < 0.05 *vs.* S913A-Ctrl. (D) A scheme summarizing the major findings of the study.

To evaluate how expression of Ulk1(S913A) affects autophagy in the mouse heart, we generated Ulk1S913A knock in (KI) mice. Interestingly, homozygous mice were not born. Thus, organ weight analyses and echocardiographic analyses were conducted with heterozygous Ulk1S913A KI and WT mice at 6 months of age. There was no statistically significant difference in organ weight, LV dimension, or cardiac function between heterozygous Ulk1(S913A) and WT mice [Tables 1 and 2]. However, the level of p62 was higher and the level of LC3-II was lower in heterozygous Ulk1S913A KI compared to WT mouse hearts [Figure 6A–C]. Four hours after chloroquine treatment, the increases in the level of LC3-II and p62 were lower in Ulk1S913A KI compared to WT mouse hearts [Figure 6A–C]. These results suggest that autophagic flux is less in Ulk1S913A KI compared to WT mouse hearts. Thus, phosphorylation of Ulk1 at Ser913 plays an important role in mediating GSK-3β-induced stimulation of autophagy in the mouse heart *in vivo*. Ulk1(S913A) mice are useful to evaluate the role of GSK-3β in mediating anti-aging effects in the heart.

## DISCUSSION

Our study shows that the age-dependent inactivation of GSK-3β that occurs through Ser9 phosphorylation promotes aging-induced cardiac dysfunction and pathological changes, including hypertrophy, fibrosis apoptosis, and senescence in the heart. Persistent activation of GSK-3β in GSK-3β KI mice prevented age-dependent cardiac dysfunction and pathological changes at 24 months of age. Although autophagy is inhibited by aging, persistent activation of GSK-3β suppresses the age-dependent decline in autophagy in the heart. GSK-3β promotes autophagy through phosphorylation of Ulk1 at Ser913, which plays an important role in mediating the anti-aging effects of GSK-3β.

The protein kinase activity of GSK-3β is negatively regulated by Ser9 phosphorylation^[29]^. Phosphorylation of GSK-3β at Ser9 was increased by aging. Together with accumulation of β-catenin and Mcl-1, proteins known to accumulate when GSK-3β is inactive^[30]^, which suggests that GSK-3β in the heart is inactivated during aging. Regulation of GSK-3β during aging appears cell type- and organ-dependent, since GSK-3β is activated in the mouse brain and neurons^[31]^. It has been shown that the activity of Akt, an important kinase responsible for Ser9 phosphorylation of GSK-3β, is increased by aging in the heart^[32]^. However, since Ser9 phosphorylation of GSK-3β is mediated by multiple kinases^[29]^, the identity of the protein kinases involved in aging-induced Ser9 phosphorylation of GSK-3β remains to be clarified.

The role of GSK-3β in aging appears complex. GSK-3β promotes aging in neuronal tissues and negatively affects lifespan in Drosophila^[33]^. On the other hand, starvation extends chronological lifespan in yeast through activation of Mck1, a GSK-3 ortholog, and activation of stress responses. Although genetic deletion of GSK-3β in mice induces embryonic lethality^[34]^, global downregulation of GSK-3α facilitates age-related pathologies in the heart, skeletal muscles, gut, liver, and skeletal systems, accompanied by mTOR-mediated suppression of autophagy^[12,35]^. Importantly, we (in this study) and others have shown that the levels of neither total nor Ser21 phosphorylated GSK-3α are altered in the heart during aging^[12]^. Thus, it is likely that inactivation of GSK-3β, but not GSK-3α, contributes to the progression of aging in the heart at baseline, whereas inactivation of GSK-3α by stress may facilitate aging in the heart. Since GSK-3α and GSK-3β have distinct functions in the heart^[23,36]^, it would be interesting to find out whether any stress facilitates inactivation of GSK-α during aging, and, if so, how inactivation of endogenous GSK-3α promotes aging in the heart and the cardiomyocytes therein.

Autophagy serves as a key mechanism for cellular quality control, and an age-dependent decline in autophagy has been established as a major driver of aging in many organisms^[14]^. Our results suggest that age-dependent inactivation of GSK-3β plays an important role in mediating the age-dependent decline in autophagy since the level of autophagy at 24 months was maintained in βKI mice. The autophagy stimulatory effect of GSK-3β in cardiomyocytes was cell-autonomous, consistent with the findings of our previous study^[37]^.

We here demonstrated that GSK-3β directly phosphorylates Ulk1, a mammalian ortholog of yeast Atg1. In fact, the level of Ulk1 phosphorylation at Ser913 was decreased in aging hearts compared to young hearts. GSK-3β-induced stimulation of autophagy is mediated through phosphorylation of Ulk1 at Ser913, as it was attenuated in the presence of Ulk1(S913A) expression. Ulk1S913A KI mice exhibited reduced levels of autophagy and autophagic flux. Recent evidence suggests that GSK-3β stimulates autophagy through phosphorylation of Ulk1 at Ser405 and Ser415 in neurons and cancer cells^[38]^. However, we could not detect Ulk1 phosphorylation in Ser405 or Ser415 in cardiomyocytes in our mass spectroscopy analyses.

The mechanism through which phosphorylation of Ulk1 at Ser913 by GSK-3β enhances autophagy is not understood at present. Ulk1 is a multidomain modulator of autophagy that contains an N-terminal kinase domain, a serine/proline-rich region, and a conserved C-terminal domain (CTD)^[39]^. The CTD is responsible for the interactions between Ulk1 and both Atg13^[40]^ and FIP200^[41]^. This domain is also involved in the translocation of Ulk1 to phagophores^[42]^. Because Ser912/3 is in the CTD, we speculate that phosphorylation of this residue by GSK-3β could affect some or all of these CTD-dependent functions of Ulk1, thereby modulating autophagy. Further experiments are needed to test this hypothesis.

Since GSK-3β has many other targets in cardiomyocytes^[29]^, we cannot formally exclude the possibility that other mechanisms mediating the effects of GSK-3β, including regulation of cardiac remodeling^[43]^ and cell survival^[34]^ through β-catenin and NF-κB, respectively, are also involved in the anti-aging effect of GSK-3β in the heart. In addition, GSK-3β inhibition may also induce aging through stimulation of cellular senescence through activation of glycogen synthase^[19]^. Thus, further experimentation is required, including creating a genetic cross between βKI mice and Ulk1S913A KI mice, to test the significance of Ulk1 Ser913 phosphorylation in mediating the anti-aging effect of GSK-3β stimulation.

There are some limitations in this study. First, due to the limited number of mice followed up for 24 months, we could not conduct chloroquine injection experiments^[44]^ with old mice for the evaluation of autophagic flux. However, our previous results show that autophagic flux is decreased in the heart by aging. The statistically significant decline in LC3-II and accumulation of p62 in WT mice at 24 months old are consistent with our previous results^[5]^. In contrast, LC3-II was increased and p62 was decreased in βKI mice at 24 months, a result directionally opposite from that observed in WT mice. Together with our observation that GSK-3β stimulates autophagy in cardiomyocytes in a cell autonomous manner, our results strongly support the notion that the ability of GSK-3β to stimulate autophagy is decreased during aging in the heart. Second, βKI and Ulk1S913A KI are both systemic and, thus, other cell types besides cardiomyocytes also express the KI mutants. Thus, we cannot formally exclude the involvement of non-myocytes in the phenotype of our KI mice. Although this study and our previous study have shown the direct cell autonomous effect of GSK-3β upon hypertrophy and autophagy in cardiomyocytes^[37,45]^, further investigation is needed to clarify the role of individual cell types in mediating cardiac aging. Finally, Ulk1S913A KI mice should be followed up longer. We expect that UlkS913A KI mice exhibit a faster decline in autophagy in the heart compared to WT mouse hears. Furthermore, a cross between βKI and Ulk1S913AKI mice would allow us to evaluate the role of Ulk1 phosphorylation at Ser913 in mediating the anti-aging effect of βKI in the heart.

In conclusion, our study suggests that inactivation of GSK-3β mediates cardiac aging in part by inactivating autophagy. Ulk1 phosphorylation at Ser913 plays an important role in mediating the autophagy-stimulatory and anti-aging effect of GSK-3β.

## Supplementary Material

Whole membrane images of WB for the s9A paper

## Figures and Tables

**Table 1.** Left ventricular weight and lung weight in Ulk1S913A knock-in mice

	WT	S913A
BW (g)	26.7 ± 1.8	29.4 ± 1.4
LVW (mg)	93.6 ± 5.1	100.9 ± 8.0
Lung (mg)	143.6 ± 6.6	150.1 ± 5.5
LVW/BW (mg/g)	3.53 ± 0.10	3.41 ± 0.14
Lung/BW (mg/g)	5.43 ± 0.17	5.15 ± 0.21
*n*	7	7

BW: Body weight; LVW: left ventricular weight; WT: wild type mice; S913A: Ulk1S913A knock-in mice.

**Table 2.** Echocardiographic measurements of Ulk1S913A knock-in mice

	WT	S913A
HR (bpm)	547 ± 8	509 ± 23
LVPWd (mm)	0.69 ± 0.03	0.67 ± 0.02
LVIDd (mm)	3.91 ± 0.05	3.98 ± 0.11
LVIDs (mm)	2.88 ± 0.13	2.89 ± 0.07
EF	0.59 ± 0.04	0.61 ± 0.02
FS	0.27 ± 0.03	0.27 ± 0.01
*n*	7	7

WT: wild type mice; S913A: Ulk1S913A knock-in mice; EF: ejection fraction; FS: fractional shortening; LVPWd: diastolic left ventricular posterior was thickness; LVIDd: diastolic left ventricular chamber diameter; LVIDs: systolic left ventricular chamber diameter.
