# Supplementary material for: Ser9 phosphorylation of GSK-3β promotes aging in the heart through suppression of autophagy
Source: J Cardiovasc Aging. Author manuscript; Available in PMC 2021 Nov 12. (PMC8589323; doi:10.20517/jca.2021.13)
Supplement: Whole membrane images of WB for the s9A paper [file NIHMS1736752-supplement-Whole_membrane_images_of_WB_for_the_s9A_paper.pdf]

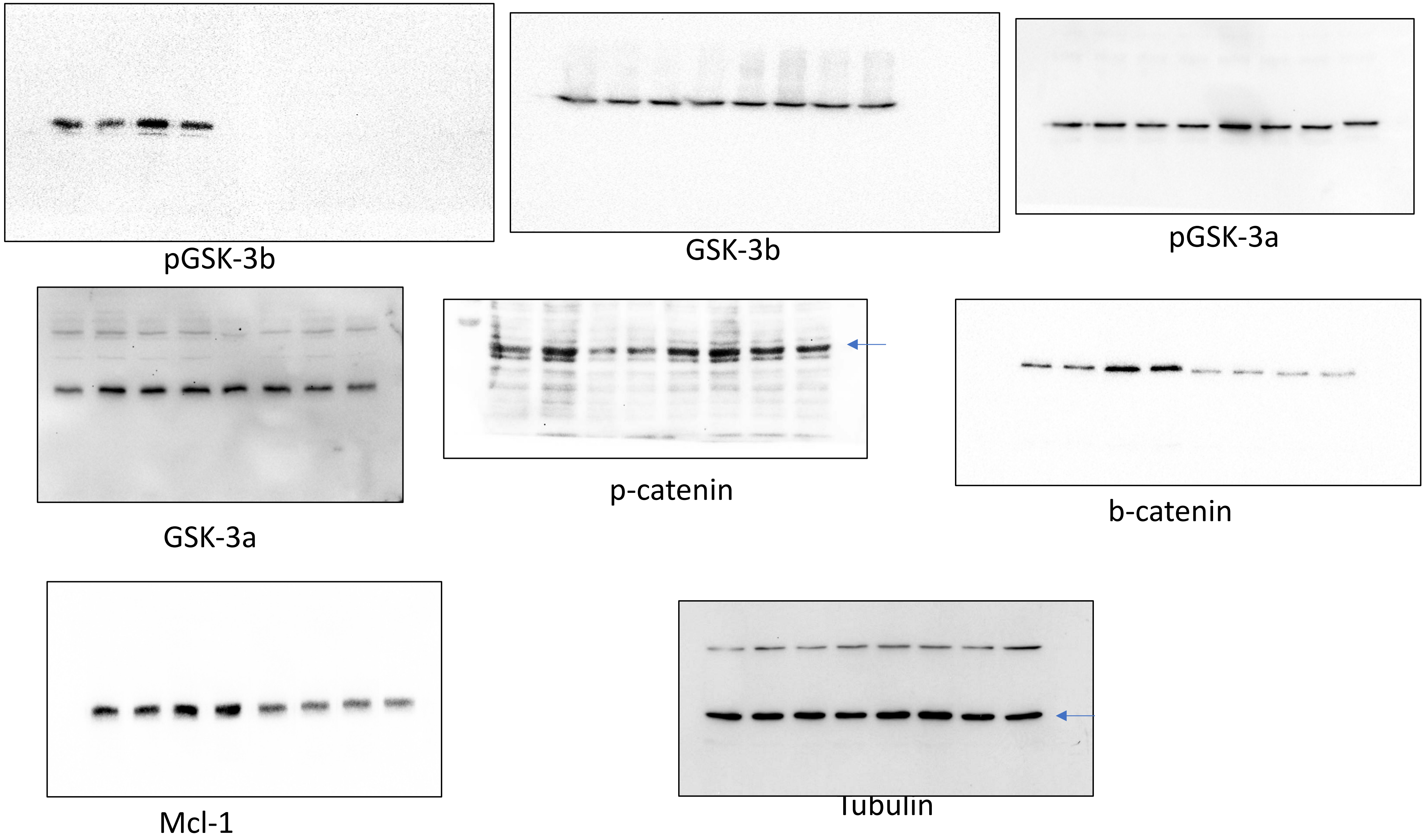

Figure 1A

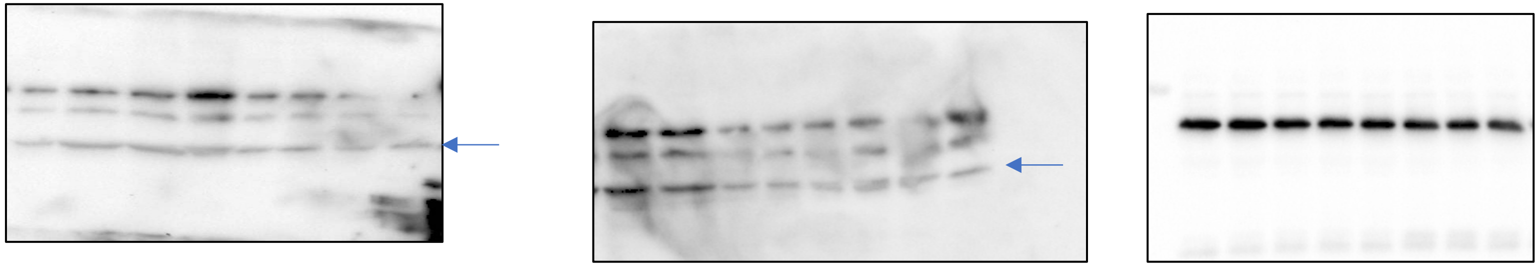

pH2AX H2AX Tubulin

Figure 3-J

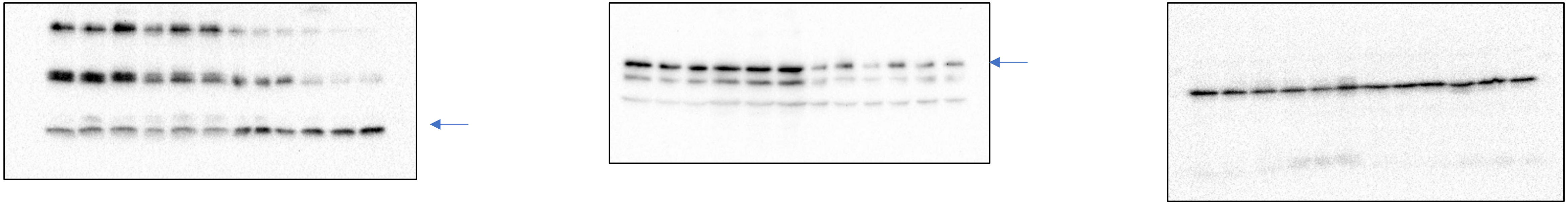

LC3

p62

Tubulin

Figure 4A

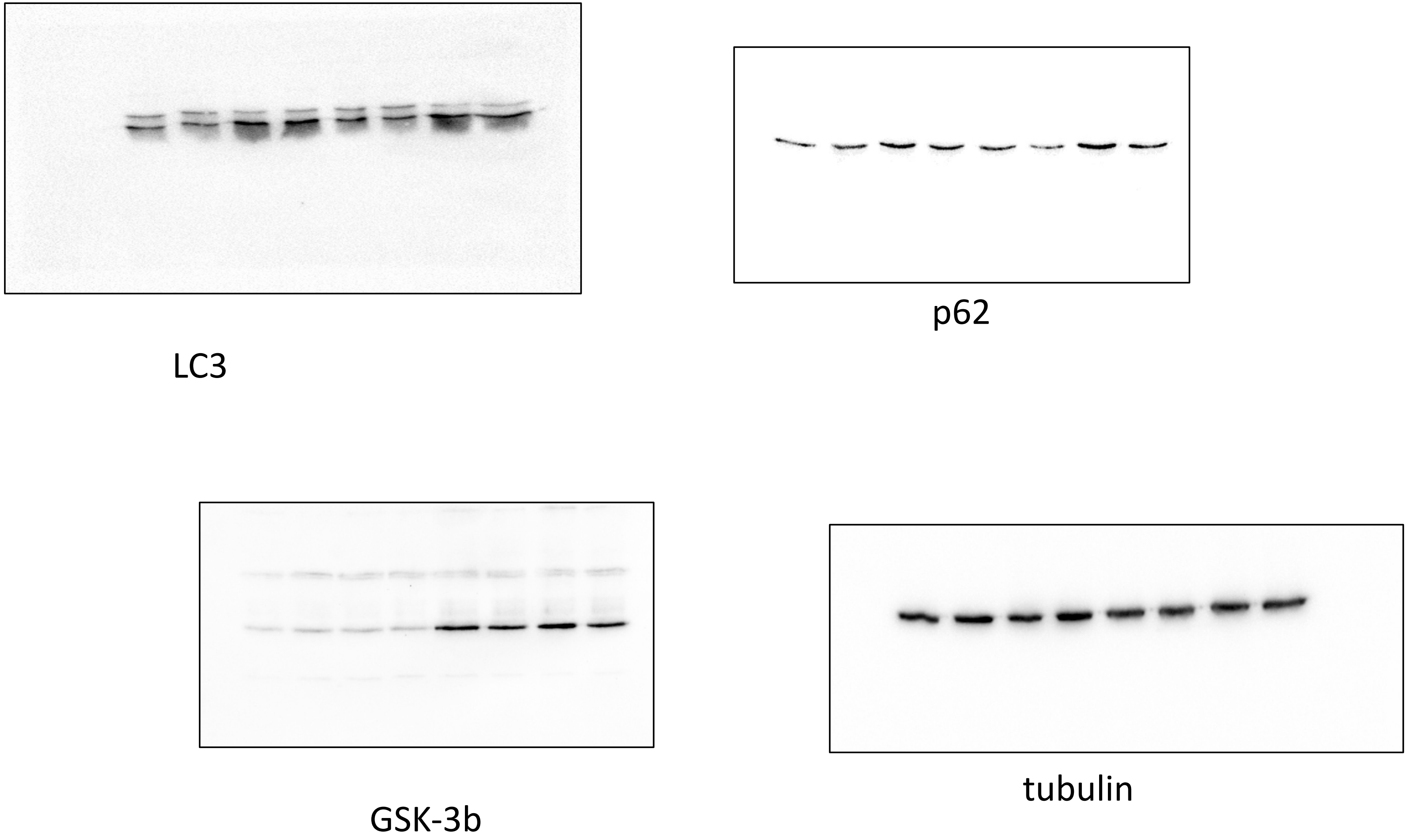

Figure 4-D

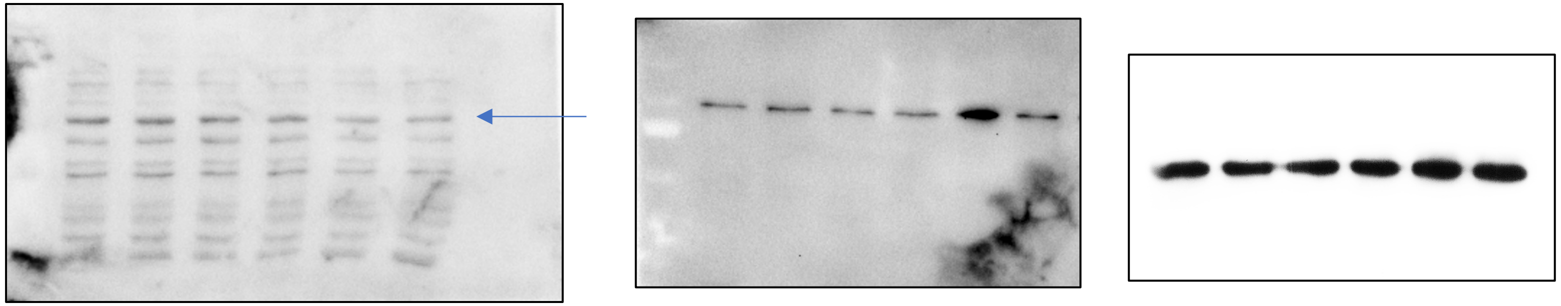

pUlk1 Ulk1 Tubulin

Figure 5-C

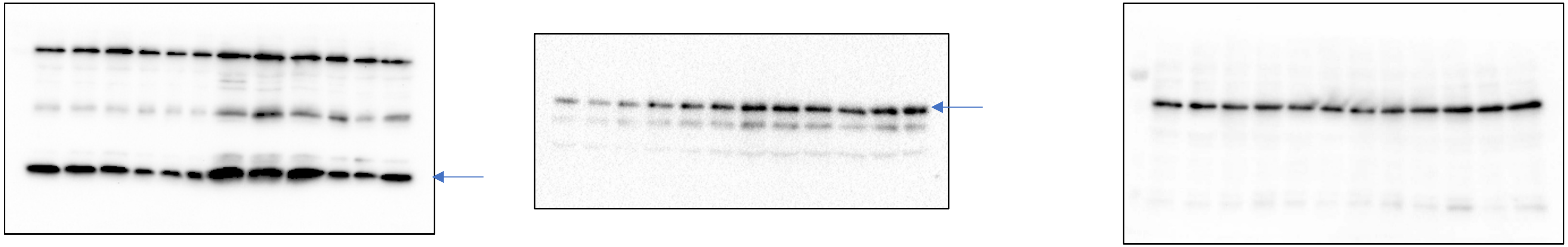

LC3 p62 tubulin

Figure 6-A
